# AtmiRNET: a web-based resource for reconstructing regulatory networks of *Arabidopsis* microRNAs

**DOI:** 10.1093/database/bav042

**Published:** 2015-05-13

**Authors:** Chia-Hung Chien, Yi-Fan Chiang-Hsieh, Yi-An Chen, Chi-Nga Chow, Nai-Yun Wu, Ping-Fu Hou, Wen-Chi Chang

**Affiliations:** College of Biosciences and Biotechnology, Institute of Tropical Plant Sciences, National Cheng Kung University, Tainan 70101, Taiwan

## Abstract

Compared with animal microRNAs (miRNAs), our limited knowledge of how miRNAs involve in significant biological processes in plants is still unclear. AtmiRNET is a novel resource geared toward plant scientists for reconstructing regulatory networks of *Arabidopsis* miRNAs. By means of highlighted miRNA studies in target recognition, functional enrichment of target genes, promoter identification and detection of *cis-* and *trans-*elements, AtmiRNET allows users to explore mechanisms of transcriptional regulation and miRNA functions in *Arabidopsis thaliana*, which are rarely investigated so far. High-throughput next-generation sequencing datasets from transcriptional start sites (TSSs)-relevant experiments as well as five core promoter elements were collected to establish the support vector machine-based prediction model for *Arabidopsis* miRNA TSSs. Then, high-confidence transcription factors participate in transcriptional regulation of *Arabidopsis* miRNAs are provided based on statistical approach. Furthermore, both experimentally verified and putative miRNA-target interactions, whose validity was supported by the correlations between the expression levels of miRNAs and their targets, are elucidated for functional enrichment analysis. The inferred regulatory networks give users an intuitive insight into the pivotal roles of *Arabidopsis* miRNAs through the crosstalk between miRNA transcriptional regulation (upstream) and miRNA-mediate (downstream) gene circuits. The valuable information that is visually oriented in AtmiRNET recruits the scant understanding of plant miRNAs and will be useful (e.g. ABA-miR167c-auxin signaling pathway) for further research.

**Database URL:** http://AtmiRNET.itps.ncku.edu.tw/

## Introduction

MicroRNAs (miRNAs) are a class of small (21–24 nucleotides) post-transcriptional gene silencers extensively found in plants and animals. Over the past decade, trends of miRNA research have been highlighted in following disciplines after the discovery of novel miRNAs. Target recognition, either computational prediction or experimental confirmation, is a dominant strategy to explore mRNAs that are hybridized by a specific miRNA. The biological protagonists of miRNA targets facilitate the inference of miRNA functions, especially for those with plenty of target genes or miRNA targets acting as hubs of genetic regulatory circuits enriched in cellular and developmental processes, biochemical secretion and pathogenesis (1, 2). Additionally, studies on transcriptional regulation of miRNA reinforce the understanding of fundamental mechanism that contributes miRNA expression (3). Not only the characteristics of miRNA genes (e.g. gene length, secondary structure and promoters) but also what transcription factors (TFs) participate in miRNA regulation under specific conditions or environments were promptly unveiled. Among them, accurately identifying miRNA promoters is an essential prerequisite for large-scale investigation of TF-miRNA regulatory relations.

**Figure 1.**
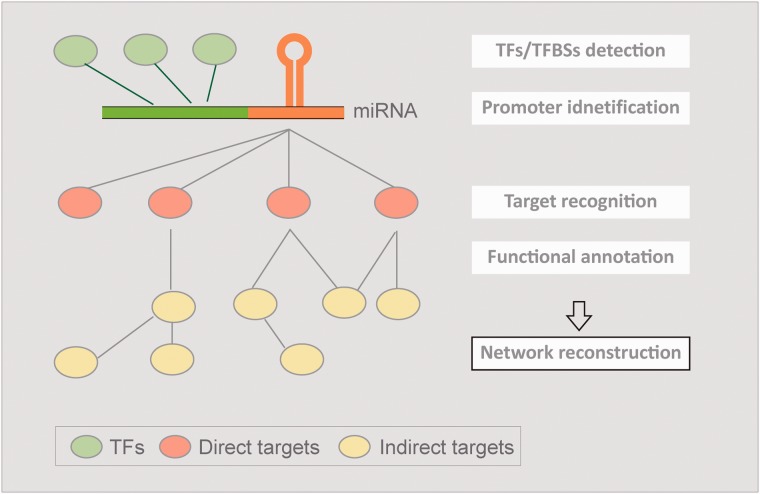
The concept of AtmiRNET. AtmiRNET summarizes the means of up-to-date miRNA research focusing on target recognition, functional enrichment of targets, promoter identification, detection of *cis-* and *trans-* elements to infer regulatory networks of *Arabidopsis* miRNAs, which are effective to augment the understanding of miRNA functions and transcriptional regulation.

Owing to the importance of up-to-date miRNA studies mentioned above for understanding regulatory mechanisms and functions of miRNAs comprehensively, this work aims to integrate these research fields and reconstruct regulatory networks of *Arabidopsis* miRNAs (Figure 1). Compared with animals, plant miRNAs are rarely investigated both in functional annotation and transcriptional regulation. *Arabidopsis thaliana* was chosen in this study due to its complete genome reference and abundant data sources. To elucidate miRNA-target interactions (MTIs) in *Arabidopsis*, we collected experimentally verified targets as well as their indirect targets, which were unprecedentedly considered. The putative targets were also provided, combining with confirmed MTIs, to perform enrichment analysis of gene ontology (GO) terms. On the other hand, the reported support vector machine (SVM)-based prediction model successfully identified miRNA transcriptional start sites (TSSs) in human (4). However, it may not be applicable for plants. To address the incompetence, we created a TSS prediction model specific to plants for identifying *Arabidopsis* miRNA TSSs. Based on the high-confidence TSSs, promoters of *Arabidopsis* miRNA were acquired to discover *cis-* and *trans-*elements using the improved concept we published previously (5). Finally, a web-based resource, AtmiRNET, was established to visualize inferred regulatory networks of *Arabidopsis* miRNAs through the integration of informative relations.

## Materials and Methods

### Establishing SVM-based model to identify *Arabidopsis* miRNA promoters

The chromosomal coordinates and annotations of 290 *Arabidopsis* pre-miRNAs were obtained from miRBase release 18 (6). The 10 kb upstream of the pre-miRNAs were retrieved for analysing putative TSSs. Moreover, experiment verified TSSs were collected from RIKEN database, which contains 12 041 unique TSSs of *Arabidopsis* protein-coding genes (7). Only genes with unique TSS were considered. Due to the lack of TSS-relevant high-throughput sequencing data on chloroplast and mitochondrion, genes locate on the chloroplast and mitochondrial DNA were excluded.

Datasets used for training promoter prediction model are summarized in Supplementary Table S2. ChIP-seq data for H3K4me3, H3K9ac and nucleosome H3 were accessed via SRA accession number SRR058388, SRR058387 and SRR052645, respectively. Additionally, TSS tags derived from sequencing of full-length cDNAs were downloaded from PlantPromDB (PPDB) (8). Bowtie was used for mapping raw reads of high-throughput sequencing datasets to *Arabidopsis* genome (9). Only unique alignments were retained for further analysis. Information of core promoter motifs were obtained from PPDB as well, including pyrimidine patch (Y Patch), initiator (Inr), CA and GA elements, and regulatory element groups (REGs) (8, 10). For the supporting evidence of TSSs, *Arabidopsis* expressed sequence tags and conservations among seven plants using phastCons method were downloaded from TAIR (11) and http://gregorylab.bio.upenn.edu/arabidopsisStructure/conservation, separately. Finally, the SVM-based model used to identify *Arabidopsis* miRNA TSSs was created by following the methodology we published previously (4). Not only the performance (sensitivity, specificity, accuracy and precision) of prediction model was evaluated by 5-fold cross-validation (CV), 63 experimentally verified miRNA TSSs from the previous study were also used for independent test (12).

### Detection of *cis*- and *trans*-regulatory elements for *Arabidopsis* miRNA genes

The coTFBS represents the common TFBS motifs coincide in promoters of a miRNA and its coexpressed annotated genes. In this work, we combined the coexpression analysis with hypergeometric *P*-values to obtain statistical significance of over-represented TFBSs in *Arabidopsis* miRNA promoters.

First, the expression profiles of *Arabidopsis* miRNAs and annotated genes were retrieved from mirEX and AtGenExpress representatively (13, 14). Since mirEX only exhibits the expression profiles of 190 miRNA precursors under 11 development stages in *Arabidopsis*, miRNAs with no expression information were discarded. For *Arabidopsis* annotated genes, developmental series of GSE5629, GSE5630, GSE5631, GSE5632, GSE5633 and GSE5634 containing 22 746 genes (probe sets) were accessed from Gene Expression Omnibus (15, 16). After the normalization process between these expression datasets, Pearson’s correlation coefficient (PCC) and Spearman’s rank correlation coefficient were applied to estimate which annotated genes are co-expressed with specific miRNAs.

Then, 1000-bp long promoters (FASTA format) of 281 *Arabidopsis* miRNA precursors were acquired according to either the experimentally verified TSSs or putative ones identified by SVM prediction model in AtmiRNET. For detecting all possible TFBSs harbored in promoter regions of miRNAs and their coexpressed annotated genes, Match program (17) was executed for searching sequence motif of TFBSs using the matrix information from TRANSFAC Spring Release 2014.1 and position weight matrices (PWMs) from the recent literature (18).

To provide reliable TF candidates for users, the frequency of coTFBSs and hypergeometric *P*-values were calculated. The formula of hypergeometric *P*-value is shown below:

$$P\left(X=k\right)=\frac{\left(\begin{array}{c} K\\ k \end{array}\right)\left(\begin{array}{c} N-K\\ n-k \end{array}\right)}{\left(\begin{array}{c} N\\ n \end{array}\right)}$$

where *N* and *n* denote the total number of TFBSs detected in promoters of all miRNA coexpressed gene groups and miRNA gene group *X*, whereas *K* and *k* represent the number of specific coTFBS detected in promoters of all miRNA coexpressed gene groups and miRNA gene group *X*, representatively. The usage of dhyper () and phyper () in R, a language and environment for statistical computing and graphics (http://www.R-project.org), were applied to obtain hypergeometric *P*-values for each coTFBS.

### Recognition of *Arabidopsis* miRNA targets and their function annotation

Putative and experimentally verified *Arabidopsis* MTIs are derived from psRNATarget and miRTarBase representatively (19, 20). The correlations between the expression levels of miRNAs and their targets are also calculated by PCC. Moreover, AtmiRNET provides the information of indirect targets, a group of genes that are indirectly mediated by a specific miRNA, that is, the downstream gene circuits of miRNA direct targets. To distinguish full downstream genes of miRNA direct targets that are experimentally verified, gene-gene interactions (edges) data were processed to perform depth-first search (DFS) algorithm, expanding all gene connections from each direct target (node) of miRNAs until no children node is found (21). For instance, Supplementary Figure S2A shows two MTIs (miR-Gene1, miR-Gene2) and five gene-gene interactions relative to a miRNA of interest. The 5 × 5 matrix in Supplementary Figure S2B was generated according to seven interactions (edges) of five elements (nodes), giving each filed value a ‘1’ if there exists the interaction between two elements, whereas a ‘0’ if no interaction. After applying DFS algorithm, an entire spanning tree was constructed (Supplementary Figure S2C) even if the regulation of feedback loop or self-control occurs. Through the above processes, entire miRNA indirect targets were distinguished.

For function annotation of *Arabidopsis* miRNA-mediated genes, the latest GO terms were collected from GO consortium (22). The hypergeometric distribution was applied to calculate the *P*-value of GO enrichment.

### Reconstructing regulatory networks of *Arabidopsis* miRNAs

By combining TF-miRNA regulatory relations, putative and experimentally confirmed MTIs, and downstream genetic circuits (indirect targets), regulatory networks of *Arabidopsis* miRNAs were reconstructed. To visualize the inferred networks, Cytoscape, an open-source software used to illustrate biomolecular interaction networks such as protein-protein, protein-DNA and genetic interactions, was applied (23).

## Results and Discussion

### Arabidopsis *miRNA promoters*

Unlike miRNAs in animals, miRNAs in plants are primarily encoded in intergenic regions and have their own promoters. According to the previous studies, most plant miRNA genes are class II genes (transcribed by RNA polymerase II), and are regulated by similar mechanisms with protein-coding genes (24). Therefore, the strategies based on chromatin structure or sequence features for promoter prediction in coding genes are useful for identifying TSSs of plant miRNAs.

**Figure 2.**
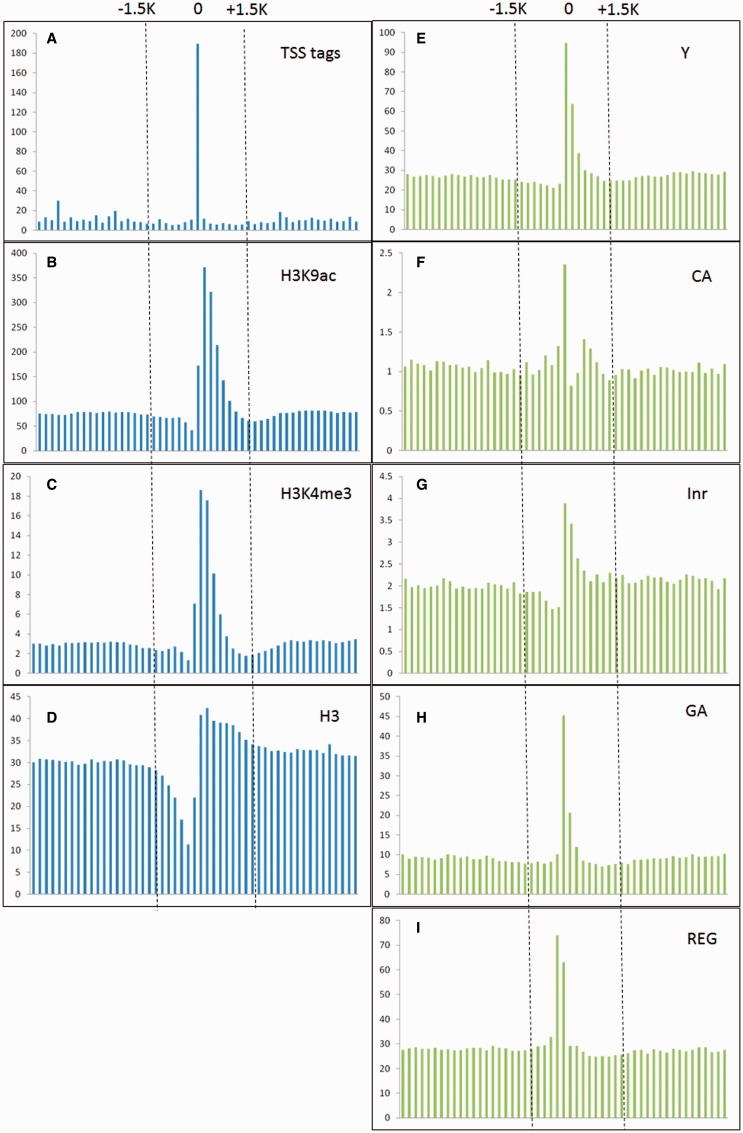
TSS tags, histone modification of H3K9ac and H3K4me3, and core promoter motifs (Y patch, CA, Inr, GA and REG) are enriched around experimentally verified TSSs, whereas nucleosome H3 are declined. The vertical and horizontal axis represent the accumulative number of each feature (X 1000 ) and genomic location (from −5 to +5 kb relative to experimentally verified TSSs, window size = 200 bp) representatively.

**Figure 3.**
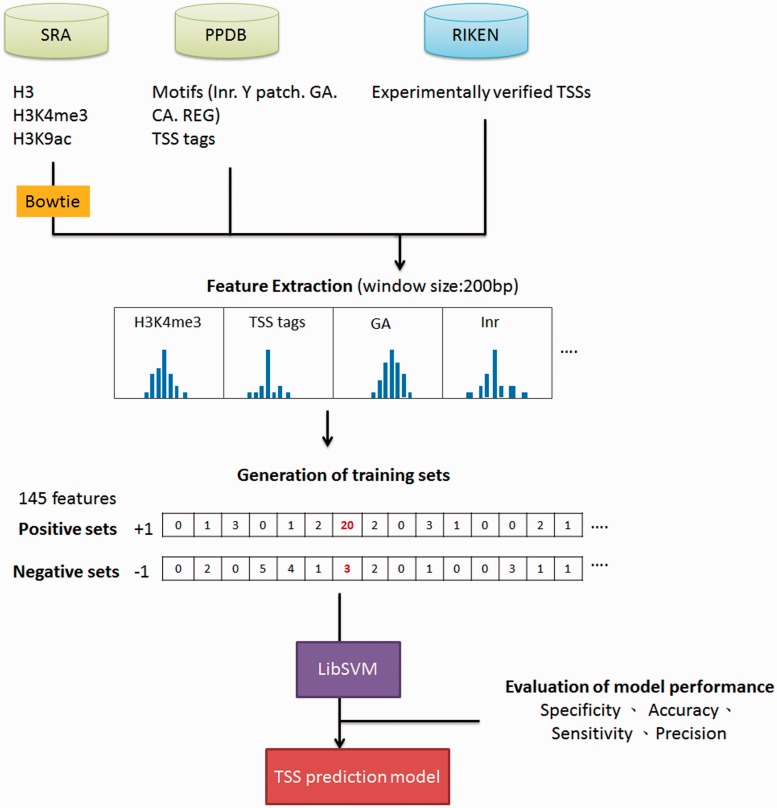
The workflow of TSS prediction model for *Arabidopsis*.

Ha *et* *al.* (25) indicated that epigenetic marks such as histone H3 lysine 4 tri-methylation (H3K4me3) and H3 lysine 9 acetylation (H3K9ac) were usually enriched around TSSs, whereas nucleosome H3 were often depleted at TSSs. Moreover, TSS tags with 5′-end of full length cDNAs are also applied to determine the 5′-boundaries of pri-miRNAs (26). Furthermore, *in silico* analysis revealed that several core promoter elements such as Inr, REG and CA elements were also positively correlated to TSS loci (27). Notably, TSS tags, histone modification of H3K9ac and H3K4me3, and five core promoter motifs are enriched around experimentally verified TSSs, whereas nucleosome H3 are declined (Figure 2). With the application of SVM, the prediction model based on above TSS-relevant datasets was created to determine *Arabidopsis* miRNA TSSs. Figure 3 summarizes the workflow of the SVM model used in AtmiRNET. Because SVM is sensitive to irrelevant and redundant data, efficient and robust feature selection is the critical process to enhance classification performance (28). To acquire the optimal features for model training, how each feature contributes to the accuracy of prediction model was estimated. Totally, 22 combinations of features derived from datasets used for model training were examined, and 5-fold CV was used to evaluate the performance of each combinatorial model. The result in Supplementary Table S1 suggests that the SVM model with all of the datasets, including TSS tags, ChIP-seq for H3, H3K9ac and H3K4me3, as well as core promoter motifs, possessed the best performance (precision: 91.28%; sensitivity: 91.23%; specificity: 91.28%; accuracy: 91.25%) and was used for further prediction of *Arabidopsis* miRNA TSSs.

**Figure 4.**
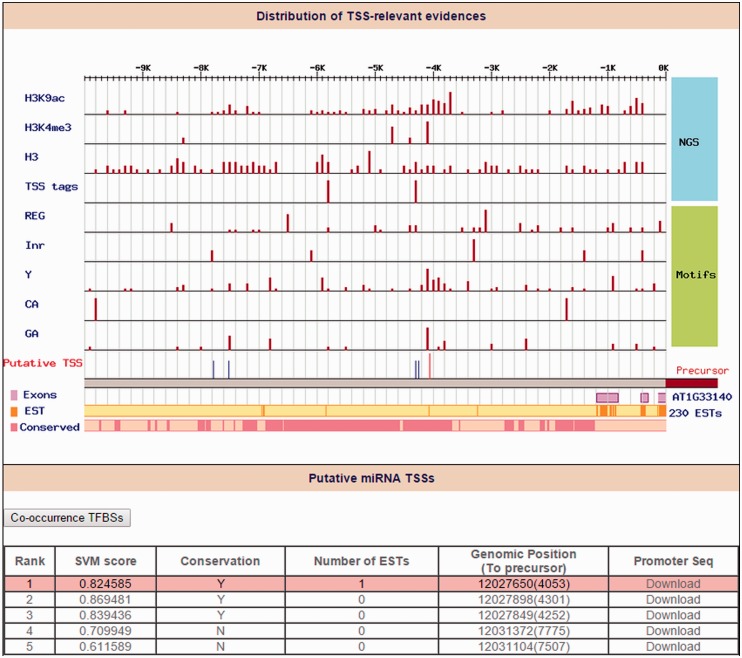
Identification of ath-miR5630b TSS. Distribution of TSS-relevant evidence (including 4 high-throughput sequencing datasets and 5 core promoter motifs) is displayed in the 10 kb upstream of the ath-miR5630b precursor. The orange line and the pink rectangles represent the initiation of expressed sequence tags (ESTs) and conservation blocks, respectively. On the other hand, the red line denotes the representative TSS for ath-miR5630b (Rank 1), and the blue lines denote other TSS candidates of ath-miR5630b.

To our current knowledge, only a limited number of miRNA promoters have been determined in plants. Although Xie *et al.* (29) reported 63 TSSs for 52 *Arabidopsis* miRNAs by 5′-RACE, most of *Arabidopsis* miRNAs TSSs remain unknown. Another drawback frequently occurs when the upstream sequences of 5′-start of miRNA precursors were retrieved as miRNA promoters for further analysis in several studies (30, 31). Rather than using ambiguous *Arabidopsis* miRNA promoters, the ‘Promoter’ function in AtmiRNET uses high-throughput sequencing datasets from TSS-relevant experiments of *A. thaliana* and plant-specific core promoter motifs enriched around TSSs to reveal the TSSs of *Arabidopsis* miRNAs. In total, 97% *Arabidopsis* miRNA TSSs (281 out of 290 miRNAs) were provided in this study. Among them, 44 miRNAs were defined as ‘intragenic’ because their pre-miRNAs embedded in the same strand of annotated genes. AtmiRNET used TSSs of host genes to represent the intragenic miRNA TSSs. By searching miRNAs of interest, users can access reliable TSSs along with the distribution of experimental evidence (an example is shown in Figure 4). The promoter sequence (5000-bp upstream from putative TSSs) of top 10 TSS candidates for each *Arabidopsis* miRNA gene can be downloaded in FASTA format.

### Putative TF-miRNA relations were explored according to the occurrence of coTFBS

Sequence-based computational approaches, e.g. PWM, are widely used to search specific TF-binding motifs within promoter regions. However, short sequence motif arising unexpected false positive rate is a critical problem. Based on the hypothesis that genes with co-expression pattern may be regulated by common TFs, the concept of coTFBSs, the common (co-occurrence) TFBS motifs coincide in promoters of a miRNA and its co-expressed annotated genes, was implemented in AtmiRNET. Annotated genes coexpressed with *Arabidopsis* miRNAs were collected to determine coincident TFBSs in their promoters. A specific TFBS located in the promoter regions of most genes in the same coexpressed group implies its corresponding TF is the most possible one controlling the expression of miRNAs. Moreover, to ensure that the over-represented TFBSs are statistically significant rather than randomly occurred, hypergeometric *P*-value for each TFBS was calculated.

**Figure 5.**
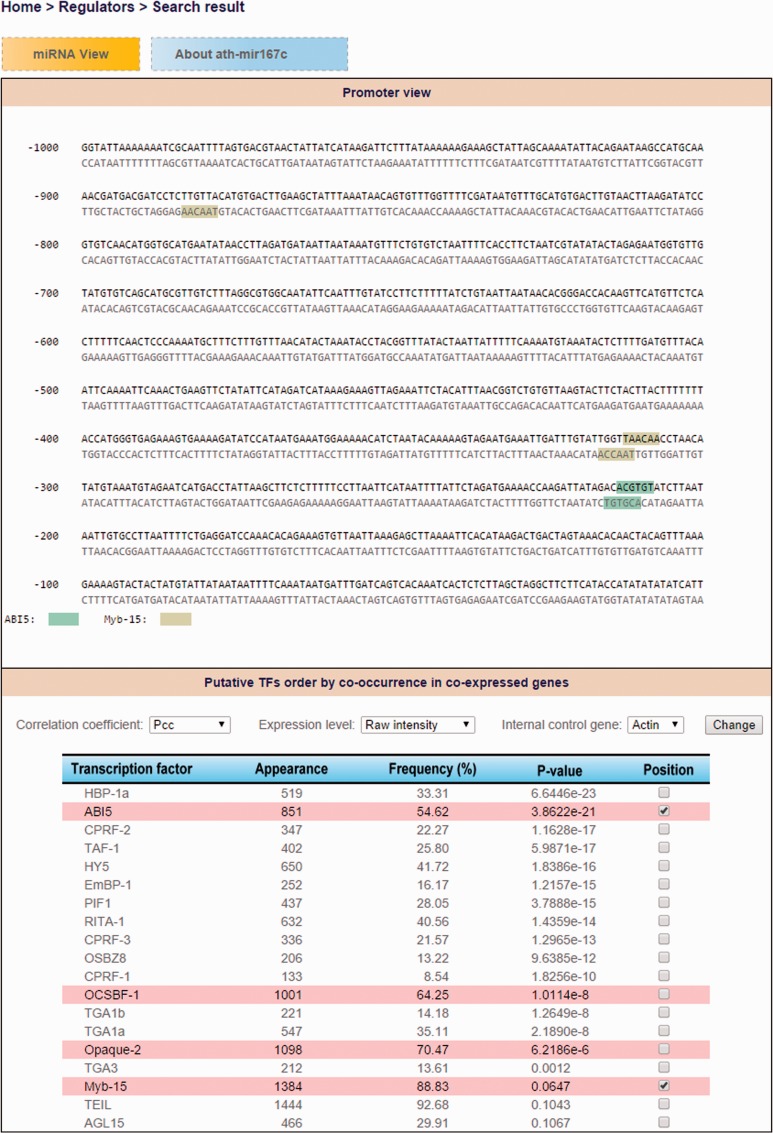
The demonstration of search result for detecting cis- and trans- elements of ath-miR167c.

Consequently, users can explore TF-miRNA regulatory relations by either miRNA search (TF candidates that regulate an *Arabidopsis* miRNA of interest) or TF search (what *Arabidopsis* miRNAs are regulated by a specific TF) by using ‘Regulator’ function in AtmiRNET. We use default (PCC, raw intensity and internal control Actin) to recommend putative TFs of *Arabidopsis* miRNAs with the frequency of coTFBS >50% and hypergeometric *P*-values <0.1. However, users are allowed to customize the parameters. Then, the binding sites and sequence motifs located in promoter regions are shown interactively by the dynamic display according to selected TF candidates. Furthermore, the co-expressed gene lists of *Arabidopsis* miRNAs are available for reference. Figure 5 demonstrates the search result for detecting *cis*- and *trans*-elements of ath-miR167c.

### Other features in AtmiRNET

**Figure 6.**
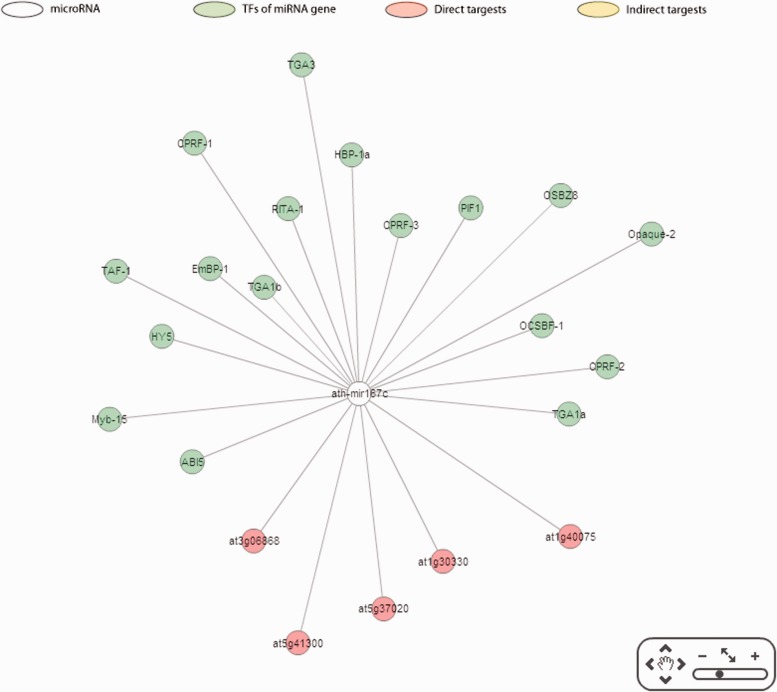
Reconstruction of miR167c regulatory network.

Apart from providing direct and indirect targets, the correlations between the expression levels of miRNAs and their targets are depicted in AtmiRNET to evaluate the validity of target prediction. Moreover, the enriched GO terms of miRNA-mediated genes allows users to deduce biological functions of miRNAs in *Arabidopsis*, which are still inadequate until now. For network reconstruction, given the graphic summary of interactions pivoted around miRNAs, users gain a perspective on the biological roles of Arabidopsis miRNAs intuitively. Figure 6 illustrates an example of inferred regulatory network in *Arabidopsis*. Users are allowed to customize the parameters for detecting *cis*- and *trans*-elements and switch to the update networks.

### Case study

#### ath-miR167c may play a crucial role in the crosstalk between ABA and auxin signaling pathways

In *A. thaliana*, plenty of evidence shows that pathways of abscisic acid (ABA) and auxin impinge on each other, suggesting the signaling crosstalk between these two plant hormones (32). ABA is believed to be related to seed development, abiotic stresses and pathogens in plants, whereas auxin, i.e. indole-3-acetic acid, is essential for numerous aspects of plant growth and development as well as environmental stresses. Rock and Sun (32) conferred possible mechanisms of crosstalk between ABA and auxin signaling pathways in roots of *Arabidopsis*; however, none of them was experimentally confirmed.

**Figure 7.**
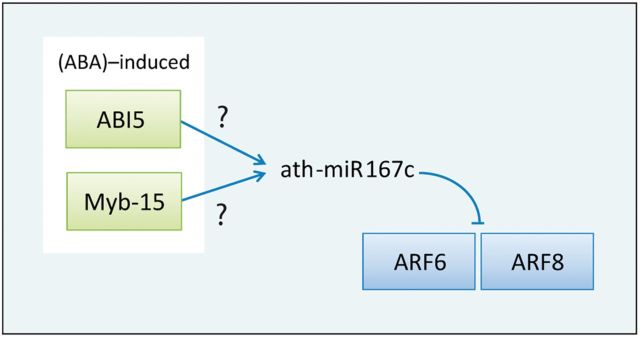
An inferred regulatory relation revolves around ath-miR167c. ABA-induced ABI5 and Myb-15 regulate miR167c, and miR167c targets ARF6 and ARF8.

Moreover, Liu *et al*. (33) surveyed the effects of three abiotic stresses including high-salinity, drought and low temperature in *Arabidopsis* and identified 14 stress-induced miRNAs by microarray data. Among them, miR167 was induced by both high salinity and drought, and miR167c was gradually increased from 2 to 24 h after exposure to high-salinity treatment. Interestingly, the result from AtmiRNET shows that two ABA-induced TFs, ABI5 and Myb-15, regulate miR167c, and two of miR167c target genes are auxin response factor 6 (ARF6) and auxin response factor 8 (ARF8) (Figure 7) (34). It reveals that miR167c may involve in stress resistance by the influence of auxin signaling pathways, and miR167c possibly connects the crosstalk between ABA and auxin signaling pathways. It is noticed that Ding *et al.* (35) suggested that transgenic overexpression of MYB15 confers enhanced sensitivity to ABA and improves drought and salt tolerance in *Arabidopsis*, which supports our finding. This important finding should be further proved.

#### Repression of ath-miR156a and miR156c triggers vegetative phase change in Arabidopsis

By using ‘Targets’ function in AtmiRNET, 13 putative targets of ath-miR156a are identified. Most of them are Squamosa promoter-binding protein-like TF family. Notably, the negative correlation of expression levels between miR156a and its targets is observed. The functional enrichment analysis of GO terms reveals that miR156a may be relative to DNA binding, development and regulation of vegetative phase change. In 2013, Yang *et al.* (36) indicated that vegetative phase change is initiated by a decrease in miR156, and miR156a and miR156c were found to play dominant roles in this transition. This evidence supports the inference of miR156a functions in AtmiRNET (also for miR156c). Supplementary Figure S1 depicts the regulatory network of miR156a consisted of its TFs, direct targets and indirect targets.

## Conclusion

Unlike animal miRNAs, our knowledge of miRNA function is comparatively insufficient in plants. Although several of bioinformatics tools or resources are geared toward plant miRNAs, it is a challenge to understand how plant miRNAs involve in significant pathways or bioreactions without reconstructing regulatory networks. In 2012, Liu *et al.* (37) developed an integrated database for *Arabidopsis* miRNA function annotations; however, the regulation of miRNA transcription was omitted. Hence, AtmiRNET is the first resource integrating TF-miRNA relations (upstream) and MTIs (downstream) of *Arabidopsis* miRNA to facilitate the reconstruction of regulatory networks and function discovery. Two case studies, the involvement of miR167c in the crosstalk between ABA and auxin-signaling pathways and miR156a in vegetative phase change, are demonstrated to reveal the potency of AtmiRNET and the fulfillment of the unmet demand in plant miRNA research.

## Supplementary Data

Supplementary data are available at *Database* Online.
